# Copy number variations and polymorphisms in HSP90AB1 and risk of systemic lupus erythematosus and efficacy of glucocorticoids

**DOI:** 10.1111/jcmm.14410

**Published:** 2019-05-24

**Authors:** Man Zhang, Yuanyuan Gu, Shunwei Huang, Qiuyue Lou, Qiaomei Xie, Zhiwei Xu, Yangfan Chen, Faming Pan, Shengqian Xu, Shengxiu Liu, Jinhui Tao, Shuang Liu, Jing Cai, Peiling Chen, Long Qian, Chunhuai Wang, Chunmei Liang, Hailiang Huang, Haifeng Pan, Hong Su, Jian Cheng, Yuzhou Zhang, Wenbiao Hu, Yanfeng Zou

**Affiliations:** ^1^ Department of Epidemiology and Biostatistics, School of Public Health Anhui Medical University Hefei China; ^2^ The Key Laboratory of Anhui Medical Autoimmune Diseases Hefei China; ^3^ School of Public Health and Social Work, Institute of Health and Biomedical Innovation Queensland University of Technology Brisbane Queensland Australia; ^4^ Department of Rheumatology and Immunology The First Affiliated Hospital of Anhui Medical University Hefei China; ^5^ Institute of Dermatology and Department of Dermatology The First Affiliated Hospital of Anhui Medical University Hefei China; ^6^ Department of Rheumatology and Immunology Anhui Medical University Affiliated Provincial Hospital Hefei China; ^7^ Department of Rheumatology and Immunology The Second Affiliated Hospital of Anhui Medical University Hefei China; ^8^ Department of Laboratory Medicine, School of Public Health Anhui Medical University Hefei China; ^9^ Department of Biochemistry and Molecular Biology, School of Basic Medicine Anhui Medical University Hefei China

**Keywords:** copy number variation, efficacy, glucocorticoids, HSP90AB1, polymorphism, systemic lupus erythematosus

## Abstract

The aim of our study was to assess the associations of HSP90AB1 copy number variations (CNVs) with systemic lupus erythematosus (SLE) risk and glucocorticoids (GCs) efficacy, as well as the relationship between HSP90AB1 single‐nucleotide polymorphisms (SNPs) and GCs efficacy. HSP90AB1 CNVs and SLE risk were analysed in 519 patients and 538 controls. Patients treated with GCs were followed up for 12 weeks and were divided into sensitive and insensitive groups to investigate the effects of CNVs (419 patients) and SNPs (457 patients) on the efficacy of GCs. Health‐related quality of life (HRQoL) was also measured by SF‐36 at baseline and week 12 to explore the relationship between CNVs/SNPs and HRQoL improvements in Chinese SLE patients. Our results indicated a statistically significant association between HSP90AB1 CNVs and SLE (*P*_BH_ = 0.039), and this association was more pronounced in the female subgroup (*P*_BH_ = 0.039). However, we did not detect association of HSP90AB1 CNVs/SNPs with efficacy of GCs. But we found a marginal association between SNP rs13296 and improvement in Role‐emotional, while this association was not strong enough to survive in the multiple testing corrections. Collectively, our findings suggest that the copy number of HSP90AB1 is associated with SLE susceptibility. But copy number and polymorphisms of HSP90AB1 may not be associated with efficacy of GCs.

## 1 INTRODUCTION

Systemic lupus erythematosus (SLE) is a systematic autoimmune inflammatory disease with a varying clinical manifestation, and its incidence is much higher in women than in men (>8:1).^1^, ^2^ In clinical practice, a first‐line therapy for SLE patients is long‐term use of glucocorticoids (GCs), which exert their biological effects after combining with glucocorticoid receptor (GR). Since the application of GCs, prognosis and quality of life of patients with SLE have been greatly improved. However, some patients fail to respond to GCs therapy, or initially respond but relapse whenever the GCs dose is reduced.^3^ Additionally, glucocorticoid‐associated adverse events are virtually universal when high doses and long‐term treatment are required.^3^ The increased risk of infection, Cushing's syndrome, femoral head necrosis, osteoporosis and other side effects affect the patients’ physical and mental health and quality of life seriously. However, the underlying mechanism of the interindividual differences in the GCs sensitivity in SLE patients is not fully understood. Our previous studies suggested that different genetic and environmental factors may contribute to the individual variation in GCs sensitivity.^4^, ^5^

Heat shock proteins 90 (HSP90) is a ubiquitously expressed molecular chaperone, as well as an important modulator of multiple innate and adaptive inflammatory processes.^6^, ^7^ The enhanced levels of HSP90 were detected in the serum of SLE patients and HSP90 deposition was found in glomeruli of some SLE patients.^2^ This suggests that HSP90 may contribute to the pathogenesis and development of SLE. In addition, as a potential treatment target for autoimmune inflammatory disease, HSP90 is crucial for GC‐GR complex, which is a necessary condition for the biological effect of GCs.^8^, ^9^ HSP90 has two major cytosolic isoforms, respectively HSP90AA1 and HSP90AB1. They play different roles in differentiation and development, as well as in response to heat stress and other environmental stimulations.^10^, ^11^ We have reported previously that genetic polymorphisms of HSP90AA1 had an influence on the response of SLE patients to GCs treatment.^12^ However, few prospective studies have examined the relationship between HSP90AB1 and GCs efficacy.

Copy number variations (CNVs) are a kind of genetic variation that changes the dosage of some disease‐related genes by deletions, duplications, and insertions.^13^ It has been suggested to contribute substantially to the genetic risk of autoimmune diseases, including SLE.^13^, ^14^ Meanwhile, evidence suggests that the region of chromosome 6p21.1, where the HSP90AB1 gene is located is associated with SLE susceptibility.^15^ This study was to confirm the role of HSP90AB1 gene CNVs with the risk of SLE and efficacy of GCs. Our previous case‐control research^16^ has investigated the association of polymorphisms in the HSP90AB1 gene with susceptibility to SLE. The results showed that the SNP rs9367190 and AG haplotype were associated with SLE susceptibility. In this study, therefore, we sought to further explore the association between HSP90AB1 gene polymorphisms and GCs efficacy. In addition, the relationship of the improvement in health‐related quality of life (HRQoL) with HSP90AB1 gene CNVs and polymorphisms was also evaluated.

## 2 MATERIALS AND METHODS

### 2.1 Subjects

In total, 519 Han SLE patients (50 males and 469 females; age: mean ± SD 35.86 ± 12.66 years) and 538 normal controls (62 males and 476 females; age: mean ± SD 35.33 ± 9.69 years) were enrolled in this study to investigate the relationship between HSP90AB1 gene CNVs and risk of SLE. No significant differences were observed in sex (*χ* ^2^ = 0.996, *P* = 0.318) and age (*t* = 0.752, *P* = 0.452) between SLE patients and controls. All subjects were recruited from the First Affiliated Hospital and the Second Affiliated Hospital of Anhui Medical University. SLE diagnosis was based on the revised criteria of American College of Rheumatology (ACR) in 1997 for SLE.^17^ For the normal controls, they did not have SLE or other autoimmune diseases, nor did they have other major diseases. Meanwhile, there was also no autoimmune disease among their immediate family members.

Four hundred and thirty‐eight patients treated with GCs were included in the analysis of CNVs and GCs efficacy. They were all followed up for 12 weeks and 419 patients completed the follow‐up. Patients who were followed had total scores of greater than 4 on the SLE disease activity index (SLEDAI). They had no exposure to GCs in three months before the follow‐up, or had maintenance treatment with low dosage of GCs. The exclusion criteria of the follow‐up survey were as follows: (a) pregnant or lactating female patients; (b) patients who required GCs plus therapy (such as lupus crisis); (c) patients who had contraindications or allergies to GCs or hydroxychloroquine therapy; (d) patients who refuse to participate in the follow‐up.

Meanwhile, a total of 476 patients (457 completed the follow‐up) were included in the analysis of single‐nucleoside polymorphisms (SNPs) and GCs efficacy. This SNPs study was conducted based on the study population of our previous case–control study,^16^ and patients in the two studies were partially overlapping. The inclusion and exclusion criteria of the current SNPs study were the same as those of CNVs survey.

The study protocol was approved by the Ethics Committee of Anhui Medical University. All participants signed the written informed consent.

### 2.2 Therapeutic regimen and efficacy appraisal

In general, at the beginning of this study, patients whose total scores on SLEDAI were <10 received GCs (prednisone) therapy of 10 mg/day to 0.5 mg/kg/day, if not (≥10), patients received GCs (prednisone) 0.5 to 1.0 mg/kg/day.^4^, ^18^ At the same time, all the patients received therapy of hydroxychloroquine. The dosage of GCs or hydroxychloroquine would be adjusted by rheumatologists dependent on the treatment effect.

We evaluated the total scores on SLEDAI of patients who participated in the follow‐up at the first week (baseline), as well as the fourth, eighth and twelfth week. The SLEDAI evaluation was administered by rheumatologists blind to genetic data. According to the total SLEDAI scores at week 12, patients were classified into two groups: insensitive group (scores ≥5) and sensitive group (scores <5). Notably, at the end of the follow‐up, patients whose scores decreased by more than 4 points were also included in the sensitive group. Patients who accepted other immunosuppressive agents due to poor responses during the therapeutic period were also considered as insensitive members.

### 2.3 HRQoL assessment

HRQoL was measured by the Medical Outcomes Study 36‐item Short Form survey (SF‐36). It is a generic instrument that is applicable to a variety of chronic diseases including SLE.^19^ The SF‐36 consists of eight subscales: physical function (PF; 10 items), role limitations due to physical problems (RP; 4 items), bodily pain (BP; 2 items), general health perceptions (GH; 5 items), vitality (VT; 4 items), social function (SF; 2 items), role limitations due to emotional problems (RE; 3 items) and perceived mental health (MH; 5 items). Scores on each subscale range from 0 to 100, and higher scores indicate better mental and physical function. In addition, the eight subscales are further summarized into the Physical Component Summary (PCS) and Mental Component Summary (MCS), which are respectively mean values of four physical subscales (PF, RP, BP, GH) and four mental subscales (VT, SF, RE, MH). HRQoL of patients was evaluated at baseline week and twelfth week, respectively. The score for the twelfth week minus the score for the baseline week was defined as an improvement score.

### 2.4 Quantitation of HSP90AB1 copy number

A custom‐by‐design Multiplex AccuCopy™ method was used for quantitation of HSP90AB1 copy number. AccuCopy™ is based on a multiplex fluorescence competitive polymerase chain reaction (PCR) assay and its basic molecular principle was illustrated by Du et al.^20^ The forward and reverse primers of target segments are presented in Table 1.

**Table 1:** The information of connection primer for HSP90AB1 gene

Chromosome	Location (Ref38 database)^a^	Amplification length (sample, competitive)^b^	Primer binding region 1	Primer binding region 2
Chr6	44253154‐44253477	352 (+0, −2)	ACTCCACCATGGGCTATATGATG	TTAAGCCATGTGAGACTTGACCAAATA

a GRCH38 reference primary assembly.

b Sample, sample DNA; Competitive, competitive DNA.

### 2.5 SNP selection and genotyping

The process of SNP selection and genotyping has been described in our previous case‐control study.^16^ Briefly, we carried out a search for all HSP90AB1 gene SNPs in the HapMap database (Chinese Han in Beijing, release No. 24/phaseII Nov08, on NCBI B36 assembly). Then, the Haploview software (version 4.2) was used to analyse the potential linkage disequilibrium. With the criteria of r^2^ >0.8 and minor allele frequency (MAF) >0.01, we finally selected two tag‐SNPs (rs9367190 and rs13296) from the total five SNPs.

Purified genomic DNA was isolated from peripheral blood by Blood Genome DNA Extraction Kit (QIAGEN, Germany). All samples had been stored at −80°C until we used. The two tag‐SNPs were genotyped by the Multiplex SNaPshot technology, which is an ABI fluorescence‐based assay allelic discrimination method (Applied Biosystems, Foster City, CA).

### 2.6 Statistical analysis

Categorical variables were presented as number (percentage) and categorical data were analysed by Chi‐square test or Fisher exact test. Continuous variables were presented as mean ± standard deviation (SD) for normally distributed or median and interquartile range (P_25_‐P_75_) for non‐normally distributed. Differences for normally distributed continuous variables were evaluated by t‐test, and the Mann‐Whitney test was for non‐normally distributed continuous variables. Univariate logistic regression analysis was applied to evaluate associations between gene CNVs/SNPs and risk of SLE and efficacy of GCs. Meanwhile, we used multivariable logistic regression to adjust for potential confounding factors.

Within this study, statistical analyses were performed by SPSS version 13.0 (SPSS, Inc, Chicago, IL). Hardy‐Weinberg equilibrium (HWE) assessed the quality of the tag‐SNPs genotype data. The haplotype analysis was conducted by on‐line software (http://analysis.bio-x.cn), and haplotype frequency <0.03 would not be listed in both insensitive group and sensitive group. *P* < 0.05 denotes statistical significance. Considering the multiple comparisons, Benjamin‐Hamburger (BH) method which regulates the false discovery rate (FDR) was applied.

## 3 RESULTS

### 3.1 The CNV genotypes and SLE risk

Frequency distribution of HSP90AB1 gene copy numbers in SLE patients and controls is summarized in Table 2. The copy numbers of HSP90AB1 ranged from 1 to 3. There was a statistically significant difference in SLE patients and normal controls (*P* = 0.003). Most patients and controls had 2 copies of the HSP90AB1 gene (97.9% of patients and 99.8% of controls). While the frequency of 1 copy was lower than 2.0%, and 3 copies were only detected in the patients’ group. We therefore combined instances of 1 copy and 3 copies in the following analyses. Copy numbers = 2 were considered normal, and copy numbers = 1 or 3 were considered abnormal.

**Table 2:** The distribution of copy number variation in HSP90AB1 gene

Copy number	Case [n (%)]	Control [n (%)]
1	5 (1.0)	1 (0.2)
2	508 (97.9)	537 (99.8)
3	6 (1.1)	0 (0)
*P* value	**0.003**	

Bold values are statistically significant (*P* < 0.05).

**Figure 1:**
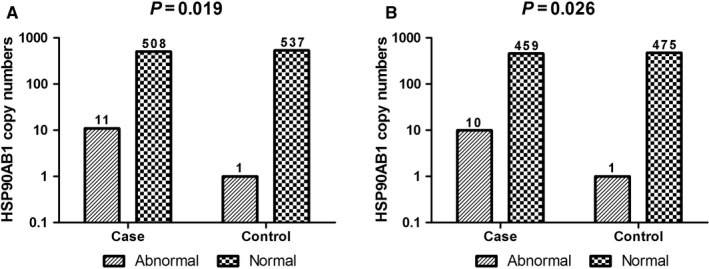
Frequency distribution of HSP90AB1 gene copy numbers in cases and controls (A and B represent all samples and females, respectively)

As shown in Table 3 and Figure 1, the HSP90AB1 gene CNVs were significantly associated with SLE (OR = 11.628, 95%CI = 1.496‐90.389, *P* = 0.019), and the association still existed after adjustment for age and sex (OR = 11.412, 95%CI = 1.467‐88.769, *P*_adj_ = 0.020, *P*_BH_ = 0.039). Since the variation of sample sizes in male subgroup was too limited, they were excluded in the stratification analysis by gender. The association between the HSP90AB1 gene CNVs and SLE was also detected in the female subgroups (OR = 10.349, 95%CI = 1.319‐81.164, *P* = 0.026, *P*_adj_ = 0.026, *P*_BH_ = 0.039). But when we attempted to explore how high (3 copies) and low (2 copies) copy numbers contribute to SLE risk, there was no association between high or low copy numbers and SLE risk (Table S1). Additionally, to explore the relationship between HSP90AB1 CNVs and clinical manifestations in SLE patients, we further compared the associations of HSP90AB1 CNVs and nephritis, arthritis, photosensitivity, serositis, skin lesions, alopecia, oral ulcers and fever, but there were no significant differences (Table S2).

**Table 3:** Association between HSP90AB1 gene CNVs and SLE risk

Copy number	Case	Control	Crude OR (95%CI)	*P*	Adjusted OR (95%CI)	*P*_adj_	*P*_BH_
All			11.628 (1.496‐90.389)	**0.019**	11.412 (1.467‐88.769)	**0.020**	**0.039**
Normal	508	537					
Abnormal	11	1					
Female			10.349 (1.319‐81.164)	**0.026**	10.352 (1.320‐81.196)	**0.026**	**0.039**
Normal	459	475					
Abnormal	10	1					

The variation of sample size in male subgroup was too limited, so they were excluded in the stratification analysis by gender.

Abbreviations: CI, confidence intervals; CNVs, copy number variations; OR, odds ratio; *P*_adj_, adjusted by age and gender; *P*_BH_, *P* value of the Benjamini‐Hochberg method for false discovery rate.

Bold values are statistically significant (*P* < 0.05).

### 3.2 The CNV genotypes and GCs efficacy

For CNVs and GCs efficacy analysis, 419 patients who completed the 12‐week follow‐up were divided into two groups, 155 in the GCs‐insensitive group and 264 in the GCs‐sensitive group (sensitive = 0, insensitive = 1). The demographic and clinical characteristics between the two groups were compared in Table 4. No significant difference was found in terms of age, gender, BMI, marital status, smoking, drinking, drinking tea, GCs exposure, baseline SLEDAI scores and dose of GCs (*P* > 0.05).

**Table 4:** Comparison of characteristics between sensitive and insensitive groups in CNVs research

Parameter	Insensitive (n = 155)	Sensitive (n = 264)	*P*
Age, years	35.40 ± 12.36	35.90 ± 12.97	0.699
Female, n (%)	136 (87.74)	242 (91.67)	0.192
BMI, kg/m^2^	21.64 ± 2.98	21.34 ± 3.39	0.357
Married, n (%)	118 (76.13)	204 (77.27)	0.789
Smoking, n (%)	8 (5.16)	13 (4.92)	0.915
Drinking, n (%)	17 (10.97)	18 (6.82)	0.138
Drinking tea, n (%)	43 (27.74)	88 (33.33)	0.233
GCs exposure before inclusion, n (%)	45 (29.03)	93 (35.23)	0.193
Baseline SLEDAI scores	11.31 ± 3.30	11.80 ± 3.66	0.175
Dose of GCs, mg/d	39.76 ± 17.27	40.22 ± 15.94	0.782

Abbreviations: CNVs, copy number variations; GCs, glucocorticoids; SLEDAI, SLE disease activity index.

As shown in Table 5, the association between HSP90AB1 CNVs and efficacy of GCs was examined. However, we could not find any association between the efficacy of GCs and HSP90AB1 gene CNVs in all patients or females. We tried to analyse the interaction between HSP90AB1 gene CNVs and SNPs in GCs efficacy, but only limited samples were available and we could not come to a conclusion.

**Table 5:** Association of HSP90AB1 gene CNVs with efficacy of glucocorticoids in SLE patients

Copy number	Insensitive	Sensitive	Crude OR (95%CI)	*P*	Adjusted OR (95%CI)	*P*_adj_	*P*_BH_
All			0.850 (0.154‐4.694)	0.852	1.077 (0.189‐6.124)	0.933	0.947
Normal	153 (98.71)	260 (98.48)					
Abnormal	2 (1.29)	4 (1.52)					
Female			0.888 (0.161‐4.913)	0.892	1.061 (0.186‐6.046)	0.947	0.947
Normal	134 (98.53)	238 (98.35)					
Abnormal	2 (1.47)	4 (1.65)				—	

The variation of sample size in the male subgroup was too limited, so they were excluded in the stratification analysis by gender.

Abbreviations: CI, confidence intervals; CNVs, copy number variation; OR, odds ratio; *P*_adj_, Adjusted by age, gender, BMI, marital status, smoking, drinking, drinking tea, GCs exposure before inclusion, baseline SLEDAI scores and GCs therapeutic dose; *P*_BH_, *P* value of Benjamini‐Hochberg method for the false discovery rate.

### 3.3 The CNV genotypes and improvement in HRQoL

To explore the relationship between HSP90AB1 gene CNVs and the improvement in HRQoL, we measured the HRQoL of 419 SLE patients, using the SF‐36 survey at baseline and week 12, respectively, and the improvement of HRQoL was calculated. However, there were no significant associations between the improvement of HRQoL and the CNVs of HSP90AB1 in SLE patients (Table S3).

### 3.4 The SNPs genotypes and GCs efficacy

The two tag‐SNPs of the HSP90AB1 gene were analysed in the 457 SLE patients who completed the follow‐up. Table 6 shows the frequencies of genotype and allele in the SLE and control groups. The genotypes of the two tag‐SNPs complied with the Hardy‐Weinberg equilibrium (*P* > 0.05).

**Table 6:** Genotype and allele frequencies of HSP90AB1 gene polymorphisms

SNP	Genotype [n (%)]			Allele [n (%)]		*P*_HWE_
	Wild	Heterozygous mutation	Homozygous mutants	Major allele	Minor allele	
rs9367190	235 (51.42)	194 (42.45)	28 (6.13)	664 (72.65)	250 (27.35)	0.145
rs13296	185 (40.48)	215 (47.05)	57 (12.47)	585 (64.00)	329 (36.00)	0.653

Abbreviations: *P*_HWE_, *P* value for Hardy‐Weinberg equilibrium; SNP, single‐nucleotide polymorphism.

After following up for 12 weeks, 186 patients were considered insensitive to GCs and 271 were considered sensitive. The demographic and clinical characteristics between sensitive and insensitive groups were compared, and the results are shown in Table S4. No significant difference was found between the two groups in terms of age, gender, BMI, marital status, smoking, drinking, drinking tea, GCs exposure, baseline SLEDAI scores and dose of GCs (*P* > 0.05).

Table 7 shows the results of univariate and multivariable logistic regression analyses of the two tag‐SNPs in the HSP90AB1 gene. We did not see a significant association between HSP90AB1 SNPs and GCs efficacy in any genetic model. Haplotype frequencies were also compared between sensitive and insensitive groups for the HSP90AB1 gene, but there were no significant differences (Table S5).

**Table 7:** Association of HSP90AB1 gene polymorphisms with efficacy of glucocorticoids in SLE patients

Models		Insensitive (n = 186)	Sensitive (n = 271)	Crude OR (95%CI)	*P*	Adjusted OR (95%CI)	*P*_adj_	*P*_BH_
rs9367190								
Dominant	CC	99 (53.23)	136 (50.18)	0.885 (0.609‐1.286)	0.523	0.907 (0.617‐1.335)	0.622	0.889
	CA + AA	87 (46.77)	135 (49.82)					
Recessive	CC + CA	178 (95.70)	251 (92.62)	0.564 (0.243‐1.309)	0.183	0.539 (0.227‐1.279)	0.161	0.889
	AA	8 (4.30)	20 (7.38)					
Additive	CC	99 (53.23)	136 (50.18)	0.847 (0.621‐1.154)	0.291	0.855 (0.622‐1.176)	0.335	0.889
	CA	79 (42.47)	115 (42.44)					
	AA	8 (4.30)	20 (7.38)					
Allele	C	277 (74.46)	387 (71.40)	0.856 (0.635‐1.154)	0.308	—	—	—
	A	95 (25.54)	155 (28.60)					
rs13296								
Dominant	GG	74 (39.78)	111 (40.96)	1.050 (0.718‐1.536)	0.802	1.028 (0.696‐1.518)	0.889	0.889
	GA + AA	112 (60.22)	160 (59.04)					
Recessive	GG + GA	161 (86.56)	239 (88.19)	1.160 (0.662‐2.030)	0.604	1.052 (0.592‐1.868)	0.864	0.889
	AA	25 (13.44)	32 (11.81)					
Additive	GG	74 (39.79)	111 (40.96)	1.064 (0.806‐1.405)	0.661	1.027 (0.774‐1.364)	0.852	0.889
	GA	87 (46.77)	128 (47.23)					
	AA	25 (13.44)	32 (11.81)					
Allele	G	235 (63.17)	350 (64.58)	1.063 (0.808‐1.398)	0.664	—	—	—
	A	137 (36.83)	192 (35.42)					

Abbreviations: CI, confidence intervals; OR, odds ratio; *P*_adj_, Adjusted by age, gender, BMI, marital status, smoking, drinking, drinking tea, GCs exposure before inclusion, baseline SLEDAI scores and GCs therapeutic dose; *P*_BH_, *P* value of Benjamini‐Hochberg method for false discovery rate.

### 3.5 The SNPs genotypes and improvement in HRQoL

After 12 weeks of glucocorticoids treatment, the improvement of HRQoL was assessed for 457 SLE patients. We found a marginal association between SNP rs13296 and the improvement in Role‐emotional (dominant model: *P* = 0.036, additive model: *P* = 0.040). However, this association was not strong enough to survive in the multiple testing corrections (dominant model: *P*_BH_ = 0.391, additive model: *P*_BH_ = 0.440). No other association was found between HSP90AB1 SNPs and the improvement of HRQoL in SLE patients (Table S6).

## 4 DISCUSSION

It is well known that SLE is a polygenic disease, a considerable number of loci have been confirmed to be associated with SLE through genome‐wide association studies of SNPs over the past several years. CNVs were also considered as critical genetic factors that can explain pathogenetic mechanism of complex diseases. Recent studies have found that genetic factors may play a role in the prognosis judgement, but such studies are mainly related to cancer. Therefore, we aimed to explore the association of HSP90AB1 gene polymorphisms and CNVs with SLE susceptibility and efficacy of GCs. In our study, polymorphisms and CNVs in the HSP90AB1 gene were found to be associated with SLE, but they have no influence on the response of SLE patients to GCs therapy.

As a member of HSP90 family, HSP90AB1 is involved in various cellular processes such as signal transduction, protein folding and morphological evolution.^21^ Recently, studies have reported up‐regulation of HSP90AB1 in many types of cancer cells, including gastric, lung and colorectal cancer, and this up‐regulation was shown to be related to the pathological grade, proliferation and poor prognosis.^21^, ^22^, ^23^, ^24^ HSP90AB1 can modulate chaperone or co‐chaperone activity and is essential for the shuttle of client proteins between cytoplasm and nucleus.^21^ It may be involved in some inflammatory diseases. Zhu et al^25^ have identified a potential role of HSP90AB1 in the genetic susceptibility to rheumatoid arthritis. However, there was very limited number of HSP90AB1 studies in other diseases including SLE.

In our previous study,^16^ we examined the genotypes and allele frequencies in the HSP90AB1 gene, and compared the haplotypes of SLE patients and normal controls. The results indicated statistically significant association between HSP90AB1 polymorphisms and SLE susceptibility. In this study, we found that HSP90AB1 gene CNVs were also a risk factor to SLE. This was the first study to demonstrate the role of HSP90AB1 gene CNVs in SLE.

Previous investigations have demonstrated the contribution of HSP90 to the inflammation and SLE progression.^2^ We speculated that HSP90AB1 gene CNVs may increase the expression of HSP90 through some mechanism, thus leading to the occurrence of SLE. CNVs can cause a coding gene dosage variation by insertion, duplication or deletion. There have been shown that CNVs influence gene expression and may account for a significant proportion of phenotypic variation.^26^ Small‐scale CNVs, on the order of a single or few copies, can lead to large‐scale changes in gene expression.^27^ By affecting gene expression via several mechanisms, CNVs may contribute to individual differences in the susceptibility to a disease.^28^ The mechanisms were apparently complex and include not only simple gene dose effects, but also insertion and deletion of gene regulatory regions, as well as changes in the physical proximity of genes and regulatory elements.^29^ On the basis of the foregoing illustration, it was possible that HSP90AB1 gene CNVs contribute to the up‐regulating the expression of HSP90 in SLE patients. However, the exact mechanisms underlying this association between the CNVs and SLE risk were still expected to be explored. And comprehensive interrogation of the functional impact of CNVs in HSP90AB1 on gene expression was warranted.

In our analysis, we found that the association between HSP90AB1 gene CNVs and SLE risk was more pronounced in female patients. Possible reason was the limited CNVs in males. So the overall effect was likely to be driven by the effect in females. Additionally, this genetic heterogeneity in gender may partly explain why the prevalence of SLE in females was much higher than in males. To some extent, our results were robust because the association between CNVs and SLE still exist after multiple corrections by FDR method. However, when instances of 1 copy and 3 copies were not combined, no association was found between high (3 copies) or low (1 copy) copy numbers and SLE risk. It may be due to the limited sample size and the low incidence of deletion and insertion variant in HSP90AB1 gene.

GCs constitute the cornerstone in the treatment of SLE, and its biological mechanism is regulated by the activation of GR.^30^ HSP90 is an important molecular chaperone and required for GR to bind the ligand and become active in vivo.^9^ However, Ouyang et al^31^ found that HSP90 expression in GC‐resistant patients was higher than that of in GC‐sensitive patients and that the increased HSP90 in GC‐resistant patients significantly tends to be intranuclear. Their study suggested that abnormal expression and distribution of HSP90 can affect the function of GR and the capacity of GCs to combine with GR.^31^ Additionally, it may affect the response of patients to synthetic GCs treatment and result in treatment failure.^32^ As noted before, CNVs in HSP90AB1 may contribute to the increased expression of HSP90. Therefore, the existence of CNVs may influence the pharmacological action of GCs through the up‐regulation of HSP90 in SLE patients. Nevertheless, no effect of HSP90AB1 gene CNVs on the efficacy of GCs was found in this study. One possible explanation was that there was a lack of coordinated chaperone interactions in the regulation of GR function. Because only purified apo GR was capable of binding a ligand with no enhancement from HSP90.^9^ HSP70 was required to work collaboratively with HSP90 to regulate and enhance GR function. Another explanation might be that the over‐expression of HSP90 caused by CNVs did not change the distribution of HSP90 in vivo, and the GC‐GR pathway was not affected. In addition, the lower frequency of HSP90AB1 gene CNVs and the limited sample size could play a role, too. Therefore, it was worthwhile to examine the association between CNVs and GCs efficiency based on a larger survey sample.

This study also showed a marginal association between HSP90AB1 SNP rs13296 and the improvement of HRQoL in Role‐emotional. However, this association was not strong enough to survive the multiple testing corrections. As an indicator of physical function, psychological state, social adaptation and a response to environmental, HRQoL can reflect the level of SLE patient's health situation.^33^ Some studies have recently pointed to the genetic basis of HRQoL. Our earlier research showed that GR gene polymorphisms were associated with the improvement of HRQoL in SLE patients treated with glucocorticoids.^18^ Similar studies had found that HRQoL was associated with some genes including IL28B,^34^ BDNF,^35^ COMT^36^ and ADRB2^37^ genes in different diseases. However, despite mounting evidence of the role of genetic factors in HRQoL, we must acknowledge that the quality of life depends on a multidimensional set of factors. Besides genetic factors, numerous factors, such as clinical manifestations and symptoms, the severity of the disease and one's personality, all of these can have an impact on a person's subjective perception of his or her health. A synthetic analysis will help us better understand the factors that affect the quality of life.

There was still some limitations in this study: (a) All samples were recruited from the First Affiliated Hospital and the Second Affiliated Hospital of Anhui Medical University, so there may be insufficient representation; (b) Hydroxychloroquine is often combined with GCs to treat SLE patients, hence, the efficacy of hydroxychloroquine to some extent may impact our results; (c) Although demographic characteristics were similar between lost patients and patients who completed the follow‐up, it was difficult to guarantee that those lost persons have no effect on the present results; (d) It is limited to the Han Chinese people and one needs more ethnic groups to strengthen the persuasion of our findings.

In summary, our study suggests that HSP90AB1 CNVs are associated with SLE susceptibility, and the association of HSP90AB1 gene polymorphisms with the HRQoL in Chinese SLE patients deserves further investigation. But HSP90AB1 CNVs and polymorphisms play no role in the efficacy of GCs.

## CONFLICT OF INTEREST

The authors declare no conflict of interests.

## AUTHOR CONTRIBUTION

Zhang M and Gu Y wrote the paper. Huang S, Lou Q, Xie Q and Chen Y followed the patients up. Xu Z and Cheng J analysed the data and prepared the tables and the figure. Pan F, Pan H, Su H and Zou Y designed the research study. Xu S, Liu S, Tao J, Liu S, Cai J and Chen P performed the research. Qian L, Wang C, Liang C and Huang H contributed the essential reagents and tools. Zhang Y, Hu W and Zou Y supervised the study. All authors reviewed and approved the final manuscript.

## DATA AVAILABILITY STATEMENT

The data that support the findings of this study are available from the corresponding author upon reasonable request.

## References

[1] Agmon‐Levin N , Mosca M , Petri M , Shoenfeld Y . Systemic lupus erythematosus one disease or many? Autoimmun Rev. 2012;11:593‐595. 22041578 10.1016/j.autrev.2011.10.020

[2] Shukla HD , Pitha PM . Role of hsp90 in systemic lupus erythematosus and its clinical relevance. Autoimmune Dis. 2012;2012:728605. 23091704 10.1155/2012/728605 PMC3471389

[3] Melo A , Melo MR , Saramago A , Demartino G , Souza B , Longui CA . Persistent glucocorticoid resistance in systemic lupus erythematosus patients during clinical remission. Genet Mol Res. 2013;12:2010‐2019. 23479142 10.4238/2013.February.19.1

[4] Zou Y‐F , Xu J‐H , Wang F , et al. Association study of glucocorticoid receptor genetic polymorphisms with efficacy of glucocorticoids in systemic lupus erythematosus: a prospective cohort study. Autoimmunity. 2013;46:531‐536. 24151836 10.3109/08916934.2013.830714

[5] Zou Y‐F , Xu J‐H , Tao J‐H , et al. Impact of environmental factors on efficacy of glucocorticoids in Chinese population with systemic lupus erythematosus. Inflammation. 2013;36:1424‐1430. 23839650 10.1007/s10753-013-9682-3

[6] Taipale M , Jarosz DF , Lindquist S . HSP90 at the hub of protein homeostasis: emerging mechanistic insights. Nat Rev Mol Cell Biol. 2010;11:515‐528. 20531426 10.1038/nrm2918

[7] Tamura Y , Torigoe T , Kutomi G , et al. New paradigm for intrinsic function of heat shock proteins as endogenous ligands in inflammation and innate immunity. Curr Mol Med. 2012;12:1198‐1206. 22804242 10.2174/156652412803306710

[8] Srivastava P . Roles of heat‐shock proteins in innate and adaptive immunity. Nat Rev Immunol. 2002;2:185‐194. 11913069 10.1038/nri749

[9] Kirschke E , Goswami D , Southworth D , Griffin P , Agard D . Glucocorticoid receptor function regulated by coordinated action of the Hsp90 and Hsp70 chaperone cycles. Cell. 2014;157:1685‐1697. 24949977 10.1016/j.cell.2014.04.038 PMC4087167

[10] Chen B , Piel WH , Gui L , Bruford E , Monteiro A . The HSP90 family of genes in the human genome: insights into their divergence and evolution. Genomics. 2005;86:627‐637. 16269234 10.1016/j.ygeno.2005.08.012

[11] Krone PH , Sass JB . HSP 90 alpha and HSP 90 beta genes are present in the zebrafish and are differentially regulated in developing embryos. Biochem Biophys Res Commun. 1994;204:746‐752. 7980538 10.1006/bbrc.1994.2522

[12] Zou YF , Xu JH , Gu YY , et al. Single nucleotide polymorphisms of HSP90AA1 gene influence response of SLE patients to glucocorticoids treatment. Springerplus. 2016;5:222. 27026916 10.1186/s40064-016-1911-4 PMC4771663

[13] Olsson LM , Holmdahl R . Copy number variation in autoimmunity–importance hidden in complexity? Eur J Immunol. 2012;42:1969‐1976. 22865047 10.1002/eji.201242601

[14] Barbosa FB , Simioni M , Wiezel C , et al. Copy number variation in the susceptibility to systemic lupus erythematosus. PLoS ONE. 2018;13:e0206683. 30485348 10.1371/journal.pone.0206683 PMC6261406

[15] Lee YH , Nath SK . Systemic lupus erythematosus susceptibility loci defined by genome scan meta‐analysis. Hum Genet. 2005;118:434‐443. 16208513 10.1007/s00439-005-0073-1

[16] Gu YY , Zou YF , Xu JH , et al. Association between polymorphism of Hsp90AB1 gene and susceptibility of systemic lupus erythematosus in Chinese population. Acta Universitatis Medicinalis Anhui. 2016;51:1151‐1155.

[17] Hochberg MC . Updating the American College of Rheumatology revised criteria for the classification of systemic lupus erythematosus. Arthritis Rheum. 1997;40:1725. 10.1002/art.1780400928 9324032

[18] Zou Y‐F , Xu J‐H , Pan F‐M , et al. Glucocorticoid receptor genetic polymorphisms is associated with improvement of health‐related quality of life in Chinese population with systemic lupus erythematosus. Clin Rheumatol. 2015;34:1537‐1544. 26255187 10.1007/s10067-015-3027-6

[19] Touma Z , Gladman DD , Ibañez D , Urowitz MB . Is there an advantage over SF‐36 with a quality of life measure that is specific to systemic lupus erythematosus? J Rheumatol. 2011;38:1898‐1905. 21724700 10.3899/jrheum.110007

[20] Du R , Lu C , Jiang Z , et al. Efficient typing of copy number variations in a segmental duplication‐mediated rearrangement hotspot using multiplex competitive amplification. J Hum Genet. 2012;57:545‐551. 22673690 10.1038/jhg.2012.66

[21] Xu C , Zhou D , Pan F , et al. A novel variant on chromosome 6p21.1 is associated with the risk of developing colorectal cancer: a two‐stage case‐control study in Han Chinese. BMC Cancer. 2016;16:807. 27756247 10.1186/s12885-016-2843-7 PMC5069896

[22] Wang H , Deng G , Ai M , et al. Hsp90ab1 stabilizes LRP5 to promote epithelial‐mesenchymal transition via activating of AKT and Wnt/beta‐catenin signaling pathways in gastric cancer progression. Oncogene. 2018;38:1489‐1507. 30305727 10.1038/s41388-018-0532-5 PMC6372478

[23] Biaoxue R , Xiling J , Shuanying Y , et al. Upregulation of Hsp90‐beta and annexin A1 correlates with poor survival and lymphatic metastasis in lung cancer patients. J Exp Clin Cancer Res. 2012;31:70. 22929401 10.1186/1756-9966-31-70 PMC3444906

[24] Haase M , Fitze G . HSP90AB1: Helping the good and the bad. Gene. 2016;575:171‐186. 26358502 10.1016/j.gene.2015.08.063 PMC5675009

[25] Zhu H , Xia W , Mo X‐B , et al. Gene‐Based Genome‐Wide Association Analysis in European and Asian Populations Identified Novel Genes for Rheumatoid Arthritis. PLoS ONE. 2016;11:e0167212. 27898717 10.1371/journal.pone.0167212 PMC5127563

[26] Freeman JL , Perry GH , Feuk L , et al. Copy number variation: new insights in genome diversity. Genome Res. 2006;16:949‐961. 16809666 10.1101/gr.3677206

[27] Mileyko Y , Joh RI , Weitz JS . Small‐scale copy number variation and large‐scale changes in gene expression. Proc Natl Acad Sci U S A. 2008;105:16659‐16664. 18946033 10.1073/pnas.0806239105 PMC2575476

[28] McCarroll SA , Altshuler DM . Copy‐number variation and association studies of human disease. Nat Genet. 2007;39:S37‐S42. 17597780 10.1038/ng2080

[29] Gamazon ER , Stranger BE . The impact of human copy number variation on gene expression. Brief Funct Genomics. 2015;14:352‐357. 25922366 10.1093/bfgp/elv017 PMC4592354

[30] Bazsó A , Szappanos Á , Patócs A , Poór G , Shoenfeld Y , Kiss E . The importance of glucocorticoid receptors in systemic lupus erythaematosus. A systematic review. Autoimmun Rev. 2015;14:349‐351. 25526806 10.1016/j.autrev.2014.12.007

[31] Ouyang J , Jiang T , Tan M , Cui Y , Li X . Abnormal expression and distribution of heat shock protein 90: potential etiologic immunoendocrine mechanism of glucocorticoid resistance in idiopathic nephrotic syndrome. Clin Vaccine Immunol. 2006;13:496‐500. 16603618 10.1128/CVI.13.4.496-500.2006 PMC1459637

[32] Picard D , Khursheed B , Garabedian MJ , Fortin MG , Lindquist S , Yamamoto KR . Reduced levels of hsp90 compromise steroid receptor action in vivo. Nature. 1990;348:166‐168. 2234079 10.1038/348166a0

[33] Thumboo J , Strand V . Health‐related quality of life in patients with systemic lupus erythematosus: an update. Ann Acad Med Singapore. 2007;36:115‐122. 17364078

[34] Matsushita H , Ikeda F , Iwasaki Y , et al. Assessment of health‐related quality of life and how it predicts the outcome of pegylated interferon and ribavirin therapy for chronic hepatitis C. J Gastroenterol Hepatol. 2014;29:337‐343. 23869873 10.1111/jgh.12337

[35] Zou YF , Wang Y , Liu P , et al. Association of BDNF Val66Met polymorphism with both baseline HRQOL scores and improvement in HRQOL scores in Chinese major depressive patients treated with fluoxetine. Hum Psychopharmacol. 2010;25:145‐152. 20196181 10.1002/hup.1099

[36] Jiménez KM , Pereira‐Morales AJ , Forero DA . Val158Met polymorphism in the COMT gene is associated with hypersomnia and mental health‐related quality of life in a Colombian sample. Neurosci Lett. 2017;644:43‐47. 28235603 10.1016/j.neulet.2017.02.050

[37] Kushnir VM , Cassell B , Gyawali CP , et al. Genetic variation in the beta‐2 adrenergic receptor (ADRB2) predicts functional gastrointestinal diagnoses and poorer health‐related quality of life. Aliment Pharmacol Ther. 2013;38:313‐323. 23786226 10.1111/apt.12378 PMC4017784

